# Effects of Threat and Motivation on Classical Musicians’ Professional Performance Practice During the COVID-19 Pandemic

**DOI:** 10.3389/fpsyg.2022.834666

**Published:** 2022-02-04

**Authors:** Guadalupe López-Íñiguez, Gary E. McPherson, Francisco J. Zarza Alzugaray

**Affiliations:** ^1^Sibelius Academy, University of the Arts Helsinki, Helsinki, Finland; ^2^Melbourne Conservatorium of Music, University of Melbourne, Melbourne, VIC, Australia; ^3^Department of Musical, Plastic and Bodily Expression, University of Zaragoza, Zaragoza, Spain

**Keywords:** basic psychological needs, COVID-19, motivation, music practice, professional musician, self-determination theory (SDT), structural equation modeling, threat

## Abstract

In the past 2 years our world has experienced huge disruptions because of COVID-19. The performing arts has not been insulated from these tumultuous events with the entire music industry being thrown into a state of instability due to the paralyzing effects of the COVID-19 pandemic. In this study, we examined how classical professional musicians’ ability to cope with uncertainty, economic struggles, and work-life interplay during COVID-19 was influenced by various factors that affect a crucial part of the development and sustainment of music careers: musicians’ practice. We analyzed responses to an online survey of 309 classical performing musicians from 41 countries in Europe and Latin America across three pandemic stages: immediately before the pandemic, during the pandemic, and when vaccines were being made available and lockdowns were being reduced or lifted. Structural equation modeling indicates relationships between perceptions of threat at the peak of the pandemic and the musicians Self- or External-Based Motivation for the three periods in which respondents were asked to reflect. Findings suggest that musicians who are more internally self-motivated seemed to be more resilient to the pandemic threats and more capable of managing their practicing routines, whereas more externally motivated musicians experienced a reduction in their dedicated time to practice during lockdown. We suggest pedagogical and policy implications, as well as future lines of research that are oriented toward supporting professional musicians in assessing and understanding their motivational drives so that they can cope with situations that disrupt their professional lives.

## Introduction

The COVID-19 pandemic has had a dramatic impact across the globe, with the music industry being significantly impacted (e.g., FIM – International Federation of Musicians, 2020). This catastrophic situation in music has been exacerbated not only by the typically uncertain situations of portfolio artists and classical musicians prior to the pandemic (e.g., Bennett, 2009; López-Íñiguez and Bennett, 2020), but also by the human alienation caused by COVID-19 through its forced (and normalized) social distancing and the closing of workplaces for music professionals. Recent reports have acknowledged how the lives of professional performing musicians across the globe have been changed because of the pandemic (e.g., Hall, 2020). The need for many Western countries to go into lockdown and close concert halls has had a severe impact on professional musicians’ financial situation, the regularity of their employment, and their sense of identity (e.g., Cohen and Ginsborg, 2021; Crosby and McKenzie, 2021; Curry, 2021; Spiro et al., 2021). Ongoing threats to their financial viability and their physical health have resulted in huge increases in stress that have impacted on the wellbeing of musicians worldwide (e.g., Schwalje and Hoffman, 2020; Spiro et al., 2021; Vance et al., 2021).

Virtual modes of music-making and music teaching have been reviewed across the pandemic as an inherent part of the life of music professionals who have been living in lockdowns. In the same way that online music teaching has experienced challenges, especially in supporting group activities (e.g., Biasutti et al., 2021; Pozo et al., 2022), online music playing has also affected social relatedness, community music making, networking, and collaboration among musicians. For example, when in-person rehearsals and concerts were canceled, researchers found that continuing artistic activities online such as by improvising asynchronously with other musicians through virtual group playing, helped reducing feelings of isolation (MacDonald et al., 2021; Margolies and Strub, 2021). Despite this, online modes of group artistic work are associated with less psychosocial benefits than in-person activities because achieving feelings of togetherness in such environments is more difficult (Draper and Dingle, 2021; Onderdijk et al., 2021). Such virtual modes of performing also pose challenges when shaping a digital offer for online audiences (Van De Werff et al., 2021). Overall, the new pandemic lifestyle has meant that many musicians have been unable to perform and rehearse in concert halls and rehearsal venues (e.g., Hall, 2020).

Despite dramatic change and reports on the ability of remote workers to manage the work-life interplay (e.g., Rocco, 2020), there are no reports of how musicians’ motivation and their practice routines have been impacted. Importantly, attempts to document how changes to the personal and professional wellbeing of classical musicians immediately before, during and when vaccines were rolled out and lockdowns were being reduced or lifted are now possible. It is for this reason that our current study examines how change occurred over time regarding the motivation of professional musicians toward their vocal/instrumental practice in a situation where the number of concerts suddenly diminished. We speculated that musicians who are more passionate about their profession might have found keeping up with their vocal/instrumental practice routines easier despite the lack of professional concert engagements. Such studies are important because professional musicians typically practice for thousands of hours to refine their craft to a level where they can gain professional employment (Ericsson et al., 1993), need to sustain their practice across their entire playing careers (e.g., McPherson et al., 2019), and are typically emotionally committed to and passionate about their performing careers (i.e., Bonneville-Roussy and Vallerand, 2020; Vallerand et al., 2022).

### Purpose of the Study

The purpose of this study was to clarify how musicians coped during the pandemic and how their professional and personal lives were impacted across this period. We were particularly interested in classically trained performing musicians from European and Latin American countries—contexts where classical music has the longest tradition—and their motivation to practice as the number of concerts dramatically decreased due to COVID-19 related cancelations.

The questions that guided our research were: to what degree across the pandemic did classical music professionals (1) sustain their practice when concerts were canceled, and (2) vary their practice according to perceptions of threat/uncertainty and motivational drives?

## Theoretical Frameworks

### Basic Psychological Needs Satisfaction: Feelings of Uncertainty and Threat During COVID-19

The study is based on the premise that professional musicians’ coping with uncertainty and economic struggles during COVID-19 will be influenced by various pandemic-related intra- and inter-individual factors that positively and negatively affect musicians’ motivation to practice. For instance, work by Spiro et al. (2021) indicates that freelance classical orchestra musicians are likely to be particularly vulnerable to threats to their wellbeing during periods of uncertainty. In this regard, the COVID-19 pandemic has been recently associated with felt insecurity and a lack of satisfaction of the basic psychological needs of people who experience a rupture in their daily lives. As an example, Vermote et al. (2021) examined the unique role and dynamic interplay of felt insecurity and satisfaction of psychological needs in the prediction of mental health on a large Belgian sample during the first 10 days of the first lockdown period. For their study on the COVID-19 pandemic these researchers adapted the *Feelings of Uncertainty and Threat Scale* by Chen et al. (2015) that was originally designed to examine if relationships between satisfaction of the psychological needs for relatedness, competence and autonomy, and well-being, would be constrained by satisfaction of the need for financial and environmental safety in South Africa. The questions included in this scale were related to threat and uncertainty in these studies and linked to the concerns about (1) the health, routines and financial situation of a person and their loved ones; (2) the overall scarcity of food and medication; and (3) the possibility for viral infection and disease in the society.

The studies by Chen et al. (2015) and Vermote et al. (2021) are based on self-determination theory (SDT; see Ryan and Deci, 2019; Evans and Ryan, 2022). SDT is a broad psychological theory of human motivation, personality development, and wellness that explains how humans are inherently oriented toward growth and wellbeing and want to feel in control and to be able to make choices. Their motivation will decrease when others make choices for them, and they feel they do not have control of their lives. In music, as in other human domains, differing contexts support the fulfillment of three basic psychological needs (i.e., autonomy, competence, and relatedness) which help sustain the quality of motivation and performance over time (Evans and Ryan, 2022).

### Self-Determination Theory: Autonomous and Controlled Motivation of Workers

In addition to the basic psychological needs introduced above, SDT (Ryan and Deci, 2019) also offers a multidimensional conceptualization of motivation that considers amotivation, intrinsic motivation, and various types of extrinsic motivation as major categories. In music, SDT has become increasingly used as a framework to study the intrinsic motivation of musicians of all ages (e.g., Evans and Bonneville-Roussy, 2016; Bonneville-Roussy et al., 2017, 2020; Freer and Evans, 2018, 2019; López-Íñiguez and McPherson, 2020; Evans and Ryan, 2022; McPherson and Hattie, 2022).

Within the theoretical underpinnings of SDT, Gagné et al. (2015) have developed and validated a SDT-motivation based scale that can be used in organizational behavior studies tackling work motivation. Using this 19-item scale, Gagné et al. (2015), have shown in large studies across seven languages and multiple countries that work motivation can be predicted by different forms of motivation, which in turn are predictably related to a variety of factors such as wellbeing, commitment, performance, and the basic psychological needs identified in SDT.

## Materials and Methods

### Design

This study utilized an *ex post* facto research design. It employed a survey to explore causal relationships between variables (i.e., feelings of uncertainty and threat, type of Self-Based/Eternal-Motivation) in real-world circumstances (i.e., average weekly accumulated practice of instrumental/vocal music across the COVID-19 pandemic stages) affecting classical performing musicians located in Europe and Latin America. The survey explored the perceptions of the musicians during three pandemic stages: immediately before the pandemic, during the pandemic, and when vaccines were being made available and lockdowns were being reduced or lifted. The musicians completed the survey during the third period where they reflected on their practices for each of the three periods examined. Our analyses used data from these three responses to (1) describe patterns of change, and (2) help establish the direction and magnitude of the identified causal relationships.

### Participants

**GRAPH 1:**
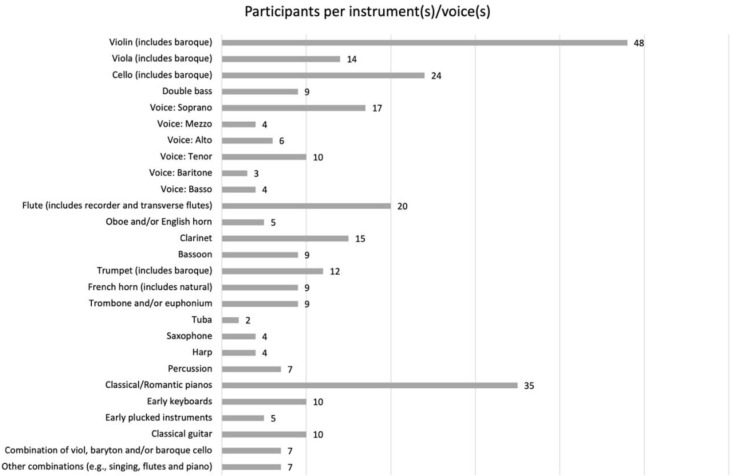
Distribution of participants per instrument(s)/voice(s).

A total of 337 classically trained, professionally active musicians took part in the study. After screening and deleting respondents who answered using the same set responses, or who were not representative of the required sample (e.g., folk/pop musicians, composers, or conductors), we performed the analyses on a total usable sample of 309 professional musicians. The sample comprised 150 (48.5%) males and 159 (51.5%) females who ranged in age from 18 to 75 years, with a mean age of 41.11 (SD = 13.30) years. The sample represents a variety of (multi-)professional roles within the music industry such as instrumental/vocal classical music soloists, chamber and orchestral musicians, ensemble and choir singers, or a combination of the above (see Graph 1). In addition, 20 of these musicians (6.47% of the participants) were combining professional performing activities with their final years of study or while pursuing a doctorate.

**GRAPH 2:**
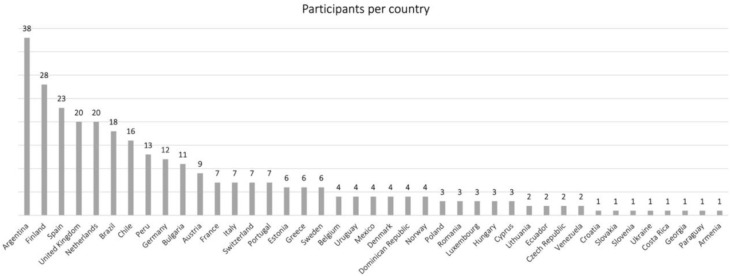
Distribution of participants per country.

We received responses from two continents, with 206 (66.7%) from Europe and 103 (33.3%) from Latin America. A total of 155 different cities were represented within 41 countries (30 countries in Europe, and 11 countries in Latin America), as shown in Graph 2. The years of professional playing ranged from 1 to 58 years, with a mean of 19.85 years (SD = 12.58). Within the survey there were also two questions addressing the amount of practice the participants reported in terms of days of practice along the week, and the typical number of hours practiced per day measured in an ordinal scale ranging from 0 to 9 points (corresponding to less than 30 min through to more than 8 h per day).

### Materials

For this investigation we used a battery of questionnaires specifically adapted to fulfill the purpose of this study. First, we used the COVID-19 adaption by Vermote et al. (2021) of the original *Feelings of Uncertainty and Threat Scale* from Chen et al. (2015) in its original version, which in this investigation showed a Cronbach’s alpha reliability index of 0.882. There were five different subscales with two questions each, including Personal Health, Financial Concerns, Unstable Situation, Supply of Sufficient Food, and Health of Loved Ones. The musicians responded by reflecting on their feelings and thoughts during the peak of the pandemic using a 7-point Likert scale (Strongly Disagree to Strongly Agree). The 10 items used in our survey were randomized in the online survey, but are shown below according to the five sources of uncertainty:

*Personal Health*
  I was concerned about my health.
  I felt threatened by a possible viral infection and disease.
*Financial Concerns*
  I was concerned about my financial situation.
  I had the feeling that my financial situation is under threat.
*Unstable Situation*
  I was concerned with how the current situation will evolve.
  I had the feeling that my daily routines were threatened.
*Supply of Sufficient Food*
  I was concerned about the scarcity of food and medication.
  I felt that our supply of food and medication is under threat.
*Health of Loved Ones*
  I was concerned about the health of my loved ones.
  I felt threatened by a possible viral infection and disease of my loved ones.

The second scale we employed was an adapted version of the *Multidimensional Motivation at Work Scale* by Gagné et al. (2015). As shown below, the scale is internally constructed by three different kinds of subscales, Amotivation (3 items), External-Based Motivation (6 items), and Self-Based Motivation (10 items). We asked Classical performing musicians to reflect on their practice habits by completing three sets of our adapted questions about how they felt about their practice immediately before, during and when vaccines were rolled out and lockdowns reduced or eliminated. The musicians responded using a 7-point Likert scale (Strongly Disagree to Strongly Agree). The 19 items used in our survey were randomized in the online survey, but are shown below according to the SDT levels of motivation:

*Amotivation*
  I didn’t practice because I really felt that I’m wasting my time at work.
  I did little practice because I didn’t think music was worth putting any effort into.
  I don’t know why I was practicing. It seemed pointless.
*Extrinsic Regulation – Social (External-Based)*
  I practiced to get others’ approval.
  I practiced because others would respect me more.
  I practiced to avoid being criticized by others.
*Extrinsic Regulation – Material (External-Based)*
  I practiced because others would reward me financially only if I put in enough effort.
  I practiced because others would offer me greater job security if I put in enough effort.
  I practiced because I risked losing my job if I didn’t put in enough effort.
*Introjected Regulation (Self-Based)*
  I practiced because I had to prove to myself that I can.
  I practiced because it made me feel proud of myself.
  I practiced because otherwise I would have felt bad about myself.
  I practiced because I personally consider it important to put effort into music.
*Identified Regulation (Self-Based)*
  I practiced because I personally consider it important to put effort into music.
  I practiced because putting effort into music aligned with my personal values.
  I practiced because music had personal significance to me.
*Intrinsic Motivation (Self-Based)*
  I practiced because I had fun.
  I practiced because it was exciting.
  I practiced because it was interesting.

The Self-Based Motivation and External-Based Motivation components of this scale were the ones we employed to build the structural equation models (SEM). The items that belonged to both subscales of the *Extrinsic Regulation (Social and Material)* were summed to construct the External-Based Motivation component. In the same way, the items that belonged to both subscales (*Introjected Regulation, Identified Regulation*, and *Intrinsic Motivation*) were summed to obtain the Self-Based Motivation component. As can be seen in Table 1, the subscales were reliable each time we gathered results, either immediately before, during or when vaccines started to be administered.

**TABLE 1:** Motivation subscales Cronbach’s alpha indexes.

	Before pandemic	During pandemic	After pandemic
External-Based Motivation	0.853	0.858	0.865
Self-Based Motivation	0.797	0.884	0.852

For the purposes of our study, the items from the original measure by Gagné et al. (2015) which responded to why people put efforts into their current job, such as “because what I do in my work is exciting” or “I don’t know why I’m doing this job, it’s pointless work,” were adapted for musicians’ instrumental/vocal practicing across the pandemic as follows: “I don’t know why I was practicing. It seemed pointless” (during the pandemic) or “I practice because it is exciting” (referring to the period when vaccines started to be administered). Thus, these items employed different verb tenses regarding the different points of data collection (before/during the pandemic, and when vaccines were administered).

The original studies by both Chen et al. (2015) also, Gagné et al. (2015) and Vermote et al. (2021) used 5-point Likert scaled responses for their measures. For the current study however, and to be consistent with the other scales in our study, we employed a 7-point Likert scale to add more discrimination among responses following recommendations by Vagias (2006).

### Average Weekly Practice and Stages of the Pandemic

As a criterion variable, we calculated what can be called the average weekly amount of practice (Practice; see Table 2) by multiplying the days per week by the number of hours per day practiced. In this sense, before the pandemic, the weekly amount of practice (Practice) was 22.86 (SD = 13.44), during the pandemic was 16.21 (SD = 15.34) and when vaccines were being rolled out was 19.41 (SD = 14.29). When comparing the three measures of Practice, statistical differences were evident in all possible combinations (see Table 2).

**TABLE 2:** Average weekly practice (Practice).

	Mean	*t*	Significant
Practice BEFORE	22.86	7.55	0.000
Practice DURING	16.21		
Practice BEFORE	22.86	4.94	0.000
Practice AFTER	19.41		
Practice DURING	16.21	–4.73	0.000
Practice AFTER	19.41		

In the analyses below, it is important to note that the stages of the pandemic correspond to:

1. Late 2019, immediately before the pandemic (BEFORE hereinafter).
2. During the peak of the pandemic between April to December 2020 (DURING hereinafter).
3. From March 2021, when vaccines were becoming available, and lockdowns were being reduced or lifted (AFTER hereinafter, as the participants responded to the survey during this stage).

### Procedure

Data collection included responses to a public online survey that was developed for large sample by adapting the validated scales outlined above and adding open-ended questions regarding their overall feelings toward their practice across the three pandemic stages. Surveys were distributed via Surveypal software in both English and Spanish. A combination of probability randomized sampling and snowball sampling was employed to access research participants in Europe and Latin America. For instance, we contacted international associations, societies, and institutions related to classical musicians’ rights, as well as colleagues and professional orchestras/choirs and ensembles to distribute our survey invitation with their networks. In addition, we arranged social media advertisements with the survey targeting the contexts of study. We kept our survey open from 14th of May till 30th of June 2021 and the approximate time to complete the survey was 15–20 min. The survey included background questions related to the participants’ age, gender, country and municipality of residence, contexts where their work took place, their professional performing profile, their years of professional experience as musicians, their employment status, and the main type of instrument/voice. We also asked about their practicing routines (days per week, time per day practiced—which was used to calculate their average per week) and concert activity (number of live/streamed concerts per month).

### Analysis of Data

Both the theoretical frameworks and the differences described above led us to continue with the statistical analysis to explore possible explanations for the existence of these differences. To study the internal relationships between the variables mentioned above, we undertook a correlational analysis to explore relationships between variables. We also ran ANOVA’s between Practice and independent variables such as sex, continent, age, and main scope of performance (regional/national/international) but in no case did we find significant relationships. Armed with this information we then moved to the most important part of our analyses where SEM were constructed to clarify not only (1) the relationships between variables but also (2) the explanatory capacity that they jointly have on the Practice in the three moments of measurement (BEFORE, DURING, and AFTER).

The main part of our analyses focused on SEM. SEM analysis creates explanatory paths among a series of different sets of data. Using this technique, it was possible to check how variables explain (or do not explain) the practice routines of the professional musicians and, at the same time, to investigate the internal direct relationships between the variables included in the paths. To explain the indirect effects—and following statistical analysis described by Khan et al. (2017)—, the *bootstrapping* technique (which involves estimating quantities about a sample by averaging estimates from multiple random data samples) was used specifying a sample of 2,000 at 95% confidence interval.

## Results

### Correlational Analysis

The correlational analysis run between the Practice and the rest of the variables (see Table 3) shows how before the pandemic, Practice was positively correlated with the Self-Based Motivation subscale (*r* = 0.259; *p* = 0.000) but was not correlated with the Threat scale (*r* = 0.087; *p* = 0.126) nor with the External-Based Motivation subscale (*r* = 0.048; *p* = 0.398).

**TABLE 3:** Correlational analysis between Practice, Threat, External-Based Motivation, and Self-Based Motivation.

	Practice BEFORE	Practice DURING	Practice AFTER
Threat	0.087	0.048	0.017
External-Based Motivation	0.048	0.081	0.105
Self-Based Motivation	0.259**	0.457**	0.350**

**p = 0.000.

During the pandemic the Self-Based Motivation subscale (*r* = 0.457; *p* = 0.000) was the only variable that was positively correlated with Practice. Both the Threat scale (*r* = −0.086; *p* = 0.132) and the External-Based Motivation subscale (*r* = 0.081; *p* = 0.158) were not correlated with Practice.

For the last period measured, Practice was correlated with the Self-Based Motivation subscale (*r* = 0.350; *p* = 0.000) but = was not correlated neither with the External-Based Motivation subscale (*r* = 0.105; *p* = 0.066) or with the Threat scale (*r* = −0.017; *p* = 0.762).

### Overall Structural Equation Models Analysis Results

We commenced our analyses from a common general model for the three-time moments studied (BEFORE, DURING, and AFTER) in which it was assumed that the internal relationships, as well as the explanatory capacity of the different variables with respect to Practice were the same for all variables. However, the data from this general model yielded statistically non-significant internal relationships, fundamentally between the Threat variable and the different Motivation variables (External-Based/Self-Based) with the criterion variable Practice. Next, the three models were adjusted, and this analysis showed that all the direct relationships between the variables were significant. Thus, these three different models provided a good fit for the data that allowed us to explain a different amount of variance of Practice in each of the time points to which the data are referred (see Table 4).

**TABLE 4:** Adjustment measures.

	BEFORE	DURING	AFTER
χ^2^	0.52	3.286	2.209
df	2	2	2
CMIN/df	0.26	1.643	1.105
*p*	0.771	0.193	0.331
CFI	1	0.989	0.997
RMSEA	0.000	0.046	0.018
AIC	16.52	19.286	18.209

Table 5 shows the variance that was explained in the models across the three periods of time that were analyzed.

**TABLE 5:** Variance explained.

	BEFORE	DURING	AFTER
External-Based Motivation	0.078	0.034	0.033
Self-Based Motivation	0.135	0.088	0.073
Practice	0.067	0.242	0.122

Tables 6, 7 show the general results of both the direct and indirect effects that are explained in detail below for the three time periods.

**TABLE 6:** Direct effects.

		BEFORE		DURING		AFTER	

		Estimate	*p*	Estimate	*p*	Estimate	*p*
External-Based Motivation	Threat	0.28	0.000	0.183	0.001	0.182	0.001
Self-Based Motivation	External-Based Motivation	0.247	0.000	0.296	0.000	0.223	0.000
Self-Based Motivation	Threat	0.211	0.000	0		0.116	0.038
Practice	Self-Based Motivation	0.259	0.000	0.476	0.000	0.35	0.000
Practice	Threat	0		−0.154	0.002	0	
Practice	External-Based Motivation	0		0		0	

**TABLE 7:** Indirect effects.

		BEFORE		DURING		AFTER	

		Estimate	*p*	Estimate	*p*	Estimate	*p*
Self-Based Motivation	Threat	0.069	0.001	0.054	0.001	0.041	0.001
Practice	Threat	0.072	0.001	0.026	0.000	0.055	0.003
Practice	External-Based Motivation	0.064	0.001	0.141	0.001	0.078	0.001

#### Results Before the Pandemic (BEFORE)

**FIGURE 1:**
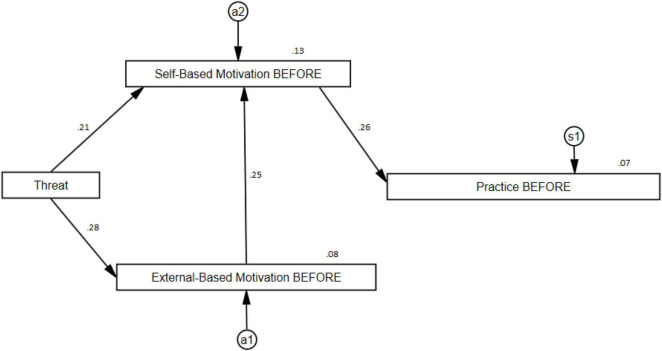
Practice path before the pandemic (BEFORE).

A first model, with the data referring to the moment before the pandemic, shows an adjusted model (CMIN/df = 0.26; *p* = 0.771; CFI = 1.0; RMSEA = 0.000) with a significant explanatory power of the PRACTICE of around 7% of the variance of it. For this, it was necessary to eliminate from the explanatory model the relationship between Threat and Practice (β_1_ = 0.024; *p* = 0.680) and between External-Based Motivation and Practice (β_1_ = −0.039; *p* = 0.505). As can be seen in Figure 1, significant relationships were obtained between External-Based Motivation and Threat (β_1_ = 0.28; *p* = 0.000), between Self-Based Motivation and External-Based Motivation (β_1_ = 0.247; *p* = 0.000), between Self-Based Motivation and Threat (β_1_ = 0.211; *p* = 0.000), and between Practice and Self-Based Motivation (β_1_ = 0.259; *p* = 0.000). All these relationships together contribute to explain around 7% of the PRACTICE’s variance. In the model it is also possible to explain around 8% of the variance of External-Based Motivation and around 13% of Self-Based Motivation.

Alternatively, if we examine the indirect effects, we can observe how they are significant in the three possible associations. Thus, Self-Based Motivation-Threat (β_2_ = 0.069; *p* = 0.001), Practice-Threat (β_2_ = 0.072; *p* = 0.001), and Practice-External-Based Motivation (β_2_ = 0.064; *p* = 0.001). Graphically, these features can be seen in Figure 1.

#### Results During the Pandemic (DURING)

**FIGURE 2:**
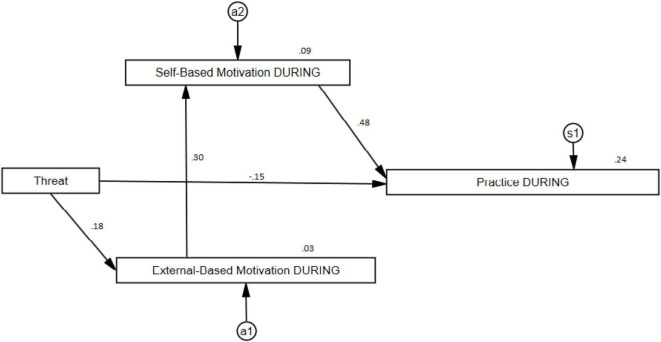
Practice path during the pandemic (DURING).

For the second model dealing with results during the pandemic, an adjusted result was also obtained (CMIN/df = 1.643; *p* = 0.193; CFI = 0.989; RMSEA = 0.046) that explains around 24% of the variance of Practice. In turn, the different associations explain 9% of the Self-Based Motivation factor and around 3% of the External-Based Motivation factor. For this, it was necessary to eliminate from the model the relationships between Threat with Self-Based Motivation (β_1_ = 0.092; *p* = 0.094) and Practice with External-Based Motivation (β_1_ = −0.037; *p* = 0.481). After these eliminations, the rest of the direct relationships between variables were all significant, as follows: External-Based Motivation and Threat (β_1_ = 0.183; *p* = 0.001), Self-Based Motivation and External-Based Motivation (β_1_ = 0.296; *p* = 0.000), Practice and Self-Based Motivation (β_1_ = 0.476; *p* = 0.000), and Threat and Practice (β_1_ = −0.154; *p* = 0.002). Again, all possible indirect relationships in the model were significant: Self-Based Motivation-Threat (β_2_ = 0.054; *p* = 0.001), Practice-Threat (β_2_ = 0.026; *p* = 0.000) and Practice-External-Based Motivation (β_2_ = 0.141; *p* = 0.001). Graphically, this can be seen in Figure 2.

#### Results When Vaccines Were Administered (AFTER)

**FIGURE 3:**
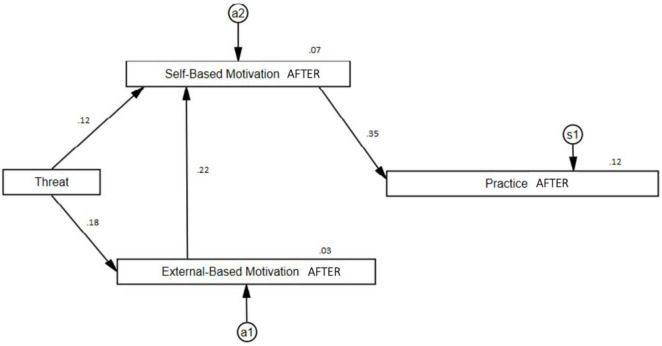
Practice path when vaccines were administered (AFTER).

Regarding the most recent results, we see how the obtained model also presents a good fit (CMIN/df = 1.105; *p* = 0.331; CFI = 0.997; RMSEA = 0.018) which explains 3.3% for External-Based Motivation factor, 7.3% for Self-Based Motivation, and 12.2% for Practice. Furthermore, as it happened with the before the pandemic model, it was necessary to eliminate from the initial general model the non-significant relationships between Threat and Practice (β_1_ = −0.079; *p* = 0.149) and between External-Based Motivation and Practice (β_1_ = −0.032; *p* = 0.560). Thus, direct significant relationships were obtained between External-Based Motivation and Threat (β_1_ = 0.18; *p* = 0.001), between Self-Based Motivation and External-Based Motivation (β_1_ = 0.223; *p* = 0.000), between Self-Based Motivation and Threat (β_1_ = 0.116; *p* = 0.038), and between Practice and Self-Based Motivation (β_1_ = 0.35; *p* = 0.000), as shown in Figure 3. Regarding indirect effects, we see how these are again maintained in all significant cases: Self-Based Motivation-Threat (β_2_ = 0.041; *p* = 0.001), Practice-Threat (β_2_ = 0.055; *p* = 0.003), and Practice-External-Based Motivation (β_2_ = 0.078; *p* = 0.001).

#### General Results

The Structural Equation path diagrams presented in this study provide an optimal fit for explaining the musicians’ Practice across the three examined time periods. For all three models, it can be noted that the musicians’ External-Based Motivation did not have a direct effect on the musicians’ Practice, but rather its effect was mediated through the musicians’ Self-Based Motivation. This means therefore that the musicians Self-Based Motivation served as a core variable, and that Threat served as a trigger to activate the musicians’ internal resources to cope with the demands required for maintaining a healthy practice schedule.

The next most important overall observations from our analyses indicate that the pattern of relationships among the paths for the first and third time-period were identical, although the strength of the path coefficients was different. For both diagrams, the Threat variable was not directly linked to the musicians’ Practice. Instead, its relationship was mediated through the musicians’ Self- and External-Based Motivations. In addition, for both models, we can observe an indirect effect of Threat through External- to Self-Based Motivation and then through the Self-Based Motivation to the musicians’ Practice.

For the middle period when the pandemic was at its peak, the flow of paths is different to the other models in two distinct ways. First, the variable Threat has a direct path to the musicians’ External-Based Motivation, which is then linked to the musicians’ Self-Based Motivation and then the musicians’ Practice. Second, Threat was directly linked to the musicians’ Practice, although unlike the other path coefficients, this path was negative.

We interpret these results in the following ways. Overall, the first and third models explain only a small percentage of variance on the musicians’ Practice (for model 1, 7%; for model 2, 12%). This result was expected in that in a normal situation (e.g., before the pandemic), the musicians’ daily lives were not driven by significant threats to their employment or their professional musical careers. During the height of the pandemic however, the impact of Threat became much more evident due to the direct negative path of Threat to Practice, and because of this path, the amount of variance increased markedly from 7 and 12% to just over 24%. In this sense, and unlike the first and third models, Threat exerted a far greater influence on the other variables that led to explaining the musicians’ Practice. An additional finding is that the variable Threat was not directly linked with the musicians Self-Based Motivation. We interpret these results as providing evidence that musicians who are more internally self-motivated are more resilient to threats, and more likely to be able to cope with situations that disrupt their professional lives such as what we have observed during the pandemic.

## Discussion and Conclusion

This study is largely exploratory in that we attempted to investigate the impact of a huge disruption in the form of COVID-19 pandemic on a group of classical professional performing musicians. In accord with work being undertaken in other areas (e.g., Vermote et al., 2021) and in music (e.g., Spiro et al., 2021), our results provide tentative evidence that the life of highly specialized artists—in this case classical musicians—can be seriously impacted during a time of great uncertainty. In our case, we tackled the understudied topic of what might help or hinder musicians in sustaining their individual practice during a pandemic, when concerts are canceled during lockdowns.

In this study, 309 professional performing musicians within the classical music industry living in European and Latin American countries displayed a variety of practice behaviors during the three stages analyzed. First, whether these musicians belonged to the Self-Based or External-Based Motivation groups did not matter in terms of the time they spent practicing immediately before the pandemic, or at the time when the vaccines were being made available. In general terms, their practice schedules during these two stages were similar, whether it was mediated by one type of motivation or the other.

Notwithstanding, at the peak of the pandemic, the musicians’ perception of threat exerted a significant impact on their practice schedules according to the two types of motivation studied. For instance, those musicians whose motivation was externally regulated, were more negatively affected at the peak of the pandemic; they displayed a maladjustment in their time spent practicing as it significantly decreased—despite this being a crucial part of professional musicianship (e.g., McPherson et al., 2019; McPherson, 2022). However, those musicians whose motivation was self-based—more internally managed—were more able to maintain their practice schedules during the peak of the pandemic despite the health and economic threats that COVID-19 posed upon them and the difficulties and challenges they experienced juggling online work and homeschooling and childcare in many cases during lockdowns (e.g., Rocco, 2020).

Overall, our results suggest that self-motivation was an important factor in the different levels of resilience/buoyancy displayed by performing musicians during the pandemic. Thus, we speculate that to survive and adapt in times of crises and uncertainty, professional musicians who have difficulties being resilient would benefit from professional support (in line with Martin and Evans, 2022). Such programs would help them develop adequate coping and stress management strategies (e.g., Jääskeläinen et al., forthcoming) as they learn to redirect their focus toward internal motives and cope during periods of uncertainty and unpredictability.

However, this study presents certain limitations, and we need to be cautious with our observations. For instance, we included a heterogeneous sample of musicians with very diverse occupational backgrounds (solo performer, chamber musician, and orchestral musician) who faced different challenges during pandemic situation. Thus, this study provides a direction for future research that can explore our results in much more detail. For instance, qualitative studies dealing with data generated from interviews or open-ended questions tackling professional performance practice might enrich these results with more nuanced explanations. Additionally, we pointed out at the beginning of the article that musicians who are passionate about their performing careers might more easily maintain their practice while dealing with a turning point in their lives such as COVID-19. For this reason, quantitative studies tackling constructs such as obsessive and harmonious passion (i.e., Bonneville-Roussy and Vallerand, 2020; Vallerand et al., 2022) might provide an additional lens to understand the tentative evidence of the influences uncovered in this study.

In terms of policy implications, we would also advocate that psychological support should be provided by organizations and government agencies for professional musicians when concerts are canceled due to lockdowns, and that these means of support should not only be adequate to provide essential needs (e.g., food and accommodation), but also in assessing musicians’ Self-Based/External-Based Motivation so that they would be aware of its impact and its relation to (1) resilience and coping, and (2) the satisfaction of their basic psychological needs.

Additionally, this study also poses implications for institutional practices which could feature transdisciplinary partnerships with others involved in the “gig” economy. For instance, career adaptability and occupational reorientation have been mechanisms successfully employed by professionals working across the creative and cultural industries (e.g., Morgan and Nelligan, 2018; López-Íñiguez and Bennett, 2020), and our results concerning the impact of COVID-19 on professional musicians’ motivation to practice similarly point toward the need of these musicians to connect and learn from/with others who work in the “gig” economy (in line with Caza, 2020) in order to develop such adaptability across their careers.

From these 309 musicians, 20 were in their final years of studies or pursuing a doctorate, as it is typical for advanced professional music students to combine work and studies. Thus, in terms of pedagogical implications, studies like ours have the potential to inform higher music education pedagogy and curriculum in terms of (1) preparedness of professional student musicians for crises, as well as on (2) the need to assess the Self-Based Motivation of aspiring students who enter institutions aimed at professionalism in music who might need to start working on resilience and coping early on.

## Data Availability Statement

Data are not available in any open data repository. A selection of the pseudonymized datasets generated for this study is available on request to the corresponding author.

## Ethics Statement

This study involves human participants and follows the ethical principles of research in the humanities and social and behavioral sciences issued by the Finnish Advisory Board on Research integrity (TENK). The study’s ethical acceptability was reviewed by the Research Ethics committee at the University of the Arts Helsinki on 19.4.2021. All participants received sufficient information about the research study and its privacy terms and provided their written informed consent to participate in this study.

## Author Contributions

GL-Í conceived the original idea of the research, applied for the ethical review of the research, prepared, published, and spread the survey internationally, and collected, screened, and translated the data from the Spanish speaking participants into English. GL-Í and GM undertook the initial designing of the study and formulated the conceptual ideas adapted in the manuscript. FZ performed all analyses with guidance on theoretical matters from GL-Í and GM. GL-Í wrote the first draft of the manuscript and received support from GM and FZ in all sections. GM provided language support, literature advice, and writing advice to GL-Í and FZ. All authors provided critical feedback at all phases, discussed the statistical analyses, contributed to the interpretation of the results, and assisted each other to complete the final version of the manuscript.

## Conflict of Interest

The authors declare that the research was conducted in the absence of any commercial or financial relationships that could be construed as a potential conflict of interest.

## Publisher’s Note

All claims expressed in this article are solely those of the authors and do not necessarily represent those of their affiliated organizations, or those of the publisher, the editors and the reviewers. Any product that may be evaluated in this article, or claim that may be made by its manufacturer, is not guaranteed or endorsed by the publisher.
